# Ways of living and working of Haitian immigrants in Western Paraná/Brazil^*^

**DOI:** 10.1590/1980-220X-REEUSP-2023-0030en

**Published:** 2023-06-26

**Authors:** Jean Bart David, Maria Lucia Frizon Rizzotto, Leda Aparecida Vanelli Nabuco de Gouvêa

**Affiliations:** 1 Universidade Estadual do Oeste do Paraná, Programa de Pós-Graduação em Saúde Pública em Região de Fronteira, Foz do Iguaçu, PR, Brazil.; 2 Universidade Estadual do Oeste do Paraná, Curso de Enfermagem, Cascavel, PR, Brazil.

**Keywords:** Emigration and Immigration, Social Determinants of Health, Health-Disease Process, Emigración e Inmigración, Determinantes Sociales de la Salud, Proceso Salud-Enfermedad, Emigração e Imigração, Determinantes Sociais da Saúde, Processo Saúde-Doença

## Abstract

**Objective:**

To analyze the migratory process, ways of living, and working conditions of Haitians residing in the municipality of Cascavel, Paraná, as well as their impact on health conditions, consumption patterns, and political and ideological life.

**Method:**

cross-sectional observational design. Data was collected through semi-structured interviews conducted from December 2019 to December 2020 with 128 participants who were Haitian immigrants residing in the municipality. Simple descriptive statistics were used for the data analysis, and the findings were discussed in conjunction with relevant literature, with the social determination theory serving as a reference.

**Results:**

The majority of participants were male 75.0% (n = 96), young adults 71.0% (n = 91), speakers of two or more languages 87.5% (n = 112), catholic 61.7% (n = 79), high school education or higher 57.0% (n = 73). They consider their life and health conditions good but are unsatisfied with the working conditions and salary.

**Conclusion:**

Haitian immigrants’ arrival facilitation to work in cold stores may indicate labor exploitation of economically and socially vulnerable groups. Immigration policies and the recognition of the educational level of their country of origin may contribute to improving the living condition of this population.

## RESUMO

**Objetivo:**

Analisar o processo migratório, os modos de vida e trabalho de haitianos que residem no município de Cascavel-Paraná e suas expressões nas condições de saúde, no padrão de consumo e na vida política e ideológica.

**Método:**

Pesquisa observacional transversal. A coleta de dados se deu por meio de entrevista semiestruturada, de dezembro de 2019 a dezembro de 2020 com 128 participantes, imigrantes haitianos residentes no município. Para análise, utilizou-se estatística descritiva simples, discutidos com a literatura pertinente, tendo como referencial a teoria da determinação social.

**Resultados:**

A maioria dos participantes foi da população masculina 75,0% (n = 96), adultos jovens 71,0% (n = 91), falam duas línguas ou mais 87,5% (n = 112), de religião católica 61,7% (n = 79), com escolaridade de nível médio ou acima 57,0% (n = 73). Consideram boas suas condições de vida e saúde, mas estão insatisfeitos com as condições de trabalho e salário.

**Conclusão:**

O favorecimento da vinda de imigrantes haitianos para atuarem em frigoríficos da região, pode indicar exploração do trabalho de grupos vulneráveis econômica e socialmente. Políticas de integração e o reconhecimento da formação escolar do país de origem, podem contribuir para melhorar a condição de vida desta população.

## RESUMEN

**Objetivo:**

Analizar el proceso migratorio, el modo de vida y el trabajo de haitianos que residen en la ciudad de Cascabel, Paraná y sus expresiones en las condiciones de salud, en las costumbres de consumo y en la vida política e ideológica.

**Método:**

Se trata de una investigación observacional transversal, con datos recopilados y sopesados con la literatura pertinente y la teoría de la determinación social. La entrevista semiestructurada se llevó a cabo con 128 participantes, inmigrantes haitianos residentes en el municipio, de diciembre de 2019 a diciembre de 2020. Para el análisis, se utilizó la estadística descriptiva simple.

**Resultados:**

La mayoría de los participantes eran hombres (75,0%) (n = 96), adultos jóvenes (71,0%) (n = 91), hablaban dos idiomas o más (87,5%) (n = 112), eran católicos (61,7%) (n = 79) y tenían estudios secundarios o terciarios (57,0%) (n = 73). Consideran que sus condiciones de vida y de salud son buenas, pero están insatisfechos con su situación laboral y su salario.

**Conclusión:**

La facilitación de la venida de inmigrantes haitianos para trabajar en frigoríficos de la región puede indicar explotación laboral de grupos económica y socialmente vulnerables. Las políticas de integración y el reconocimiento de la escolaridad del país de origen pueden contribuir a mejorar las condiciones de vida de este grupo de inmigrantes.

## INTRODUCTION

International immigration has been growing due to natural disasters, economic and social problems, internal conflicts, and political instability that obligate people to leave their countries of origin searching for protection, work, and better living conditions. The search for stability, better work opportunities, and salary that allow the accomplishment of personal or family life projects are the main variables that influence the decision to migrate by individuals or groups of people^(1,2)^. The fragile Haitian economic situation, the natural disasters, and their political history have been determinants for many young people to opt for immigration to countries with higher job offers, previously France and the United States of America, and in the last decade, Latin-American countries such as Brazil.

In the case of Haitian immigration to Brazil, the Brazilian participation in the United Nations Stabilization Mission in Haiti (2004 to 2017) was decisive, and the earthquake occurred in 2010, associated with the economic and social development of Brazil in the first decade of the XXI century. According to data from International Migration Observatory (OBMigra), 106.1 thousand Haitians entered Brazil from 2011 to 2018, and although they observed a decrease in the number of registers of Haitian immigrants in the last years, from January to August of 2019, there were 10,682 applications for registration, and in the same period in 2020 more 4,339 requests^(3,4)^. The mechanisms of immigration and stay of Haitians in Brazil have been occurring mainly by “humanitarian visa,” created in 2012 for Haitian immigrants and, later, for Syrian and Venezuelan as a response to emergent situations introducing amendments in the legislation recognizing immigrant rights^(5)^.

The entry of the first Haitians occurred mainly by land, Acre State, but with the humanitarian visa, it became by air, through São Paulo city, with later displacement to smaller municipalities where there were industrial poles, mainly poultry and pork cold stores. The Cascavel/Paraná city, by the characteristics of its economy based on agribusiness, with big slaughterhouses of poultry and pork for exportation, constituted an important destination for Haitian immigrants searching for jobs, being among the 10 Brazilian cities with the most regularly registered immigrants^(6)^. The restricted relation with the reproductive chain of cold stores reveals a closing of other productive sectors for Haitians, who submit themselves to this type of job because it is the only alternative to labor insertion, not being properly a choice but an imposition by their condition of being an immigrant of a peripheral country^(7)^.

The demand for this type of activity is directed especially towards young male workers, which explains that only 20.0% of the total Haitian immigrants with permission to reside in Brazil are women, although this relationship has been changing in recent years due to the increase in visas for family reunions^(8)^. The difficulties confronted by women immigrants are higher than men, given the preference of companies for young workers of the male sex. Haitian women, as immigrants and black people, are socially vulnerable once they are admitted to more precarious workplaces and with lower salaries^(9)^.

Although studies^(10,11)^ had been conducted with Haitian immigrants residents in Brazil, the delimitation of the present research aims, based on the critical epidemiology and the social determination theory^(12)^, to analyze the migratory process, the ways of living and working of Haitians that reside in the municipality of Cascavel-Paraná, and their expressions in health conditions, consumption pattern, and political and ideological life.

## METHODS

### Study Design

Cross-sectional observational study.

### Site

The study was conducted among the Haitian community residing in the municipality of Cascavel, Paraná, Brazil.

### Selection Criteria

The study included Haitian immigrants who had permanent residence in the municipality of Cascavel, Paraná, for at least six months and were at least 18 years old. Only those who agreed to participate in the research were included, while immigrants who were passing by, visiting family members, or did not have a valid visa to stay in Brazil were excluded.

### Sample Definition

The convenience sample for this study comprised 128 subjects, with 32 women (25.0%) and 96 men (75.0%). The sample included individuals who had entered the country at different times and represented various local Haitian communities.

### Data Collection

We collected the data through interviews using a semi- structured script from December 2019 to December 2020. Due to the SARS-CoV-2 pandemic, we conducted the data collection in two formats: in-person and Computer Assisted Telephone Interviewing (CATI). In the in-person format, we visited two Haitian communities in the town and interviewed 86 subjects at their residences. Through CATI, we interviewed 42 subjects who were identified based on the recommendations of participants from the in-person interviews. All interviews, regardless of format, were conducted individually and lasted an average of 40 minutes. Prior authorization was obtained for audio recording, and interviews were scheduled in advance. We translated the interview script into the Creole language and gathered data on identification, socioeconomic factors, daily life, consumption patterns, ideological beliefs, policies, recreation, health, and immigration. The first author, who is of Haitian origin, conducted all the interviews, which facilitated communication and the overall development of the study.

### Analysis and Data Treatment

We transcribed and recorded the objective data in an Excel spreadsheet. The data were organized into tables, categorized according to the dimensions of the critical process matrix proposed by Breilh^(12)^. We analyzed the data using simple descriptive statistics and discussed the findings in relation to relevant literature, with the social determination theory as our reference framework^(12)^.

### Ethical Aspects

The project was approved by the Research Ethics Committee (REC) with opinion number 3.535.040 in 2019, and an addendum with opinion number 4.021.535 in 2020. All participants provided their informed consent by signing the Informed Consent Form (ICF) to participate in the study. We strictly adhered to the guidelines outlined in resolution #466/2012 and n510/2016, which govern research involving human subjects. During the CATI data collection, we digitally sent the ICF to participants, and after ensuring their understanding of the terms presented by the interviewer, we obtained verbal authorization before collecting their signed consent form.

## RESULTS

Table 1 presents the sociodemographic profile of the 128 participants in the study. The majority of participants were male, accounting for 75.0% (n = 96), while 25.0% (n = 32) were female. In terms of age distribution, 71.0% (n = 91) fell within the 26–40 age range, with a notable concentration of participants aged 31–35, accounting for 32.8% (n = 42) of the total. On the other hand, the lowest proportion was observed among participants aged 46–50, comprising only 4.7% (n = 6) of the sample. Regarding marital status, 42.2% (n = 54) reported being in a stable union, and 37.5% (n = 48) indicated being married. In terms of educational attainment, 57.0% (n = 73) of participants had completed or partially completed high school. Additionally, a significant proportion of participants, 87.5% (n = 112), reported speaking two or more languages, including French, Creole, and Portuguese.

**Table 1.** Number and percentage of Haitian immigrants, according to sex, marital status, age, language and educational level. Cascavel, Paraná, Brazil, 2020.

Variables	N	%
**Sex**		
Male	96	75.0
Female	32	25.0
**Marital status**		
Married	48	37.5
Single	17	13.3
Separated/divorced	6	4.7
Widowed	3	2.3
Stable union	54	42.2
**Age**		
18 to 25 year old	17	13.3
26 to 30 year old	27	21.0
31 to 35 year old	42	32.8
36 to 40 year old	22	17.2
41 to 45 year old	14	11.0
46 to 50 year old	6	4.7
**Language**		
1 language	16	12.5
2 languages or more	112	87.5
**Educational level**		
Middle School complete/unc.	37	28.9
High school complete/unc.	73	57.0
Technical school	13	10.1
University education	5	4.0

Table 2 provides insights into the migratory process of the participants. It reveals that the influx of immigrants to Brazil began in 2012, with the majority, 57.8% (n = 74), arriving between 2015 and 2017. Among the participants, 96.9% (n = 124) chose air travel as their mode of transportation, and 70.3% (n = 90) had previously resided in another city before settling in Cascavel, Paraná. Of those, 35.2% (n = 45) initially lived in São Paulo. In terms of the motivation behind their migration, 43.7% (n = 56) initiated the process independently, while 28.1% (n = 36) received assistance from family members. Regarding their previous occupations in their home country, 40.6% (n = 52) worked in trade, 25.0% (n = 32) were engaged in agriculture, and interestingly, 6.2% (n = 8) were high school teachers. The living conditions in Brazil were considered good by 67.9% (n = 87) of the respondents, while 32.1% (n = 41) expressed dissatisfaction and deemed their living conditions in the city as poor. Notably, all interviewees mentioned job stability as the primary reason for choosing Cascavel as their place of residence.

**Table 2.** Number and percentage of Haitians, according to the 1^st^ city of residence, way of entrance, year of arrival, migration process, and work carried out in the origin country and evaluation regarding their living condition in Brazil. Cascavel, Paraná, Brazil, 2020.

Variables	N	%
**1** ^**st**^ **city of residence**		
São Paulo	45	35.2
Rio de Janeiro	8	6.3
Acre	4	3.1
Cascavel	38	29.7
Foz do Iguaçu	8	6.2
Toledo	12	9.4
Porto Alegre	4	3.1
Joinville	6	4.7
Curitiba	3	2.3
**Way of entrance**		
Land border	4	3.1
Airplane	124	96.9
**Year of arrival**		
2012	4	3.1
2013	9	7.1
2014	11	8.6
2015	32	25
2016	17	13.3
2017	25	19.5
2018	14	10.9
2019	13	10.2
2020	3	2.3
**Migration process**		
Own initiative	56	43.7
Brazilian government program	12	9.4
Businessmen invitation	5	3.9
With family help	36	28.1
Other way	19	14.8
**Work carried out in the origin country**		
Trading	52	40.6
Agriculture	32	25
Student	13	10.2
Did not work	12	9.4
Driver	9	7.0
Teacher/High school	8	6.2
Street vendor	2	1.6
**Evaluation regarding their living condition in Brazil.**		
Good	87	67.9
Bad	41	32.1

Table 3 presents data regarding the current employment status of Haitian immigrants. Out of the participants, 82.0% (n = 105) claimed to be employed, and 73.6% (n = 93) reported being knowledgeable about labor rights in Brazil. Among those who are employed, 91.4% (n = 96) have a formal employment relationship governed by the Consolidation of Labor Laws (CLT), while 8.6% (n = 9) are self-employed.

**Table 3.** Number and percentage of Haitians, according to work sector, work station, training, transport to work, daily hour of work, time at work, income, work environment, and job satisfaction. Cascavel, Paraná, Brazil, 2020.

Variables	N	%
**Employment relationship**		
*CLT*	96	75.0
Self-employed	9	7.0
Do not work	23	18.0
**Knowledge about labor rights**		
Yes	93	73.6
No	35	27.4
**Work sector**		
Industry slaughterhouses/por	76	72.4
Civil construction	17	16.2
Service sector	12	11.4
**Work stations**		
Cuttin	64	61.0
Washing	12	11.4
Bricklayer auxiliary	15	14.3
Bricklayer	2	1.9
Housekeeper	3	2.9
Driver/apps	9	8.5
**Training**		
Yes	36	28.1
No	92	71.9
**Transportation to work**		
Bus	88	83.8
Bike	8	7.6
Own car	3	2.8
By foot	6	5.8
**Working hour/day**		
8h	78	74.3
More than 8h	27	25.7
**Time at work**		
3 months or more	11	10.5
1 to 2 years	41	39.0
3 years or more	53	50.5
**Income**		
1 minimum wage	97	92.4
Up to two minimum wage	8	7.6
**Work environment**		
Excellent	2	1.9
Good	8	7.6
Regular	12	11.4
Bad	83	79.1
**Work satisfaction**		
Satisfied	7	6.7
Unsatisfied	98	93.3

The primary occupation for 72.4% (n = 76) of the participants is working in poultry and pork cold stores, followed by 16.2% (n = 17) involved in civil construction, mainly as auxiliary bricklayers (14.3%, n = 15). In slaughterhouses, 61.0% (n = 64) are engaged in cutting tasks, and 11.4% (n = 12) are involved in washing activities. Regarding job training, 71.9% (n = 92) did not receive any prior training for their current job. Furthermore, 25.7% (n = 27) work more than 8 hours per day, and the income for the vast majority, 92.4% (n = 97), amounts to a monthly minimum wage. The majority of participants, 89.5% (n = 94), have been working for 1 to 3 years or more. Public transport is the primary means of transportation for 83.8% (n = 88) of the respondents. In terms of the work environment and job satisfaction, the results indicate that 79.1% (n = 83) perceive the work environment as poor, and 93.3% (n = 98) express dissatisfaction with their current job.

Table 4 presents aspects related to the health of the interviewed immigrants. When asked about their response to health problems, 40.6% (n = 52) reported resorting to hospitals, while 29.7% (n = 38) sought Basic Care Units (UBS). Only 10.2% (n = 13) indicated self-medication as their approach, although the use of pharmacies by 19.5% (n = 25) could be considered a form of self-medication. The majority, 72.7% (n = 93), claimed not to use any medication. In terms of physical activity, 67.1% (n = 86) reported not engaging in any form of physical activity. Additionally, 52.3% (n = 67) did not take preventive measures for their health. When evaluating the quality of their nutrition, 52.3% (n = 67) considered it healthy or very healthy, while 28.9% (n = 37) had a different perception. Regarding their overall health condition, 72.7% (n = 93) assessed it as good, while 27.0% (n = 35) described it as bad. Furthermore, 19.5% (n = 25) had to take sick leave due to illness, and 5.5% (n = 7) had been on sick leave for more than six months.

**Table 4.** Number and percentage of Haitians, according to how they address health issues, use of medicine, practice of physical activities, health prevention, nutrition, health status, sick leave, SUS evaluation and access to social programs. Cascavel, Paraná, Brazil, 2020.

Variables	N^o^	%
**Health issues**		
Self-medication	13	10.2
Pharmacy	25	19.5
Basic Care Unit	38	29.7
Hospital	52	40.6
**Use/Medication**		
Paracetamol	13	10.1
Other/not specified	22	17.2
Do not use	93	72.7
**Physical activity**		
GYM	5	3.9
Sports	12	9.4
Biking	25	19.6
Do not practice	86	67.1
**Health prevention**		
Immunization	28	21.8
Medical appointments	33	25.9
No	67	52.3
**Nutrition evaluation**		
Very healthy	37	28.9
Healthy	67	52.3
Unhealthy	24	18.8
**Health status**		
Good	93	72.7
Bad	35	27.3
**Sick leave**		
1 to 6 months	18	14.0
More than 6 months	7	5.5
Never	103	80.5
**SUS evaluation**		
Excellent	94	73.4
Good	19	14.8
Bad	6	4.7
Terrible	9	7.1
**Social programs access**		
Yes	1	1.0
No	127	99

Table 5 presents information on Haitian immigrants’ consumption life, daily life, political life, ideology, and recreation. It reveals that 100.0% of the interviewed immigrants reside in rented accommodations, with the majority (57.8%, n = 74) consisting of 4 to 5 rooms. The households typically have 3 to 4 people (57.0%, n = 73). In terms of expenses, for the majority of participants (74.2%, n = 95), the main expenditure is their day-to-day living in Brazil, including rent and food. On the other hand, 25.8% (n = 33) of the participants reported using a significant portion of their income to send remittances to family members back in their home country. During difficult times, 67.2% (n = 86) mentioned relying on support from friends.

**Table 5.** Number and percentage of Haitians, according to the type of residence, number of rooms, number of residents, main expenses, access to public education, access to public health, religion, support/economic, discrimination, and recreation. Cascavel, Paraná, Brazil, 2020.

Variables	N	%
**Type of residence**		
Rented	128	100
**N. of rooms**		
1 to 2 rooms	16	12.5
2 to 3 rooms	38	29.7
4 to 5 rooms	74	57.8
**N. of people living at home**		
1 to 2 residents	37	28.9
3 to 4 residents	73	57.0
5 to 6 residents	18	14.1
**Main expenses**		
Remittances	33	25.8
Rent/Food	95	74.2
**Access to Public Education**		
Yes	3	2.3
No	125	97.7
**Access to public Health**		
Yes	116	90.6
No	12	9.4
**Religion**		
Protestant	33	25.8
Catholic	79	61.7
Did not answered	16	12.5
**Economic/Support**		
Relatives	27	21.1
Friends	86	67.2
Did not answered	15	11.7
**Discrimination**		
Yes	17	13.3
No	111	86.7
**Recreation**		
Watching TV	74	57.8
Talking to friends	37	29.0
Music	17	13.2
**Membership in trade unions**		
Yes		
No	128	100

Regarding access to services, only 2.3% (n = 3) mentioned having access to public education, while 90.6% (n = 116) stated having access to public health services. In terms of religious affiliation, 61.7% (n = 79) identified as Catholic. As for recreation, 57.8% (n = 74) indicated television as their primary recreational activity. In terms of experiences in Brazil, the majority (86.7%, n = 111) stated that they have not faced discrimination. Furthermore, 100% of the participants reported not being involved in trade unions or political associations.

## DISCUSSION

The immigration of Haitians to Brazil, although having particular institutional support, was preceded by difficulties in the country of origin, with a lack of circulation of information regarding the mechanisms of visa application, which caused some of them to utilize coyote networks and illegal routes to enter the country through land borders. The observed aspect such as the good level of education of Haitian immigrants, which 71.0% (n = 91) completed high school or higher education (technical school and university education), and 87.5% (n = 112) speak two or more languages do not guarantee an insertion on the market that corresponds to their level of education. A study in another locality of Brazil^(13)^ also identified similar difficulties, such as access to workstations in the formal market, besides challenges in the social and cultural context. Associated with that, we highlight the frustrations of personal projects caused by low salaries, the type of work carried out, besides the absence of inclusion policies that favored the integration, and the confrontation of the discrimination, many times non-verbal but manifested through gestures, looks, and non-verbal expressions. The difficulties faced by Haitian immigrants in the local society integration, labor activities, or prejudice they suffer are similar to the difficulties faced by the Brazilian black population victim of the existent structural racism.

The insertion into the formal market does not guarantee security to immigrants once the sector of the slaughterhouse industry presents high rotativity of labor to decrease the eventual costs with labor issues, as well as the workers themselves search for other options given the major dissatisfaction that this type of job entails. The work in slaughterhouse industries for Brazilian workers such as immigrants does not favor professional realization nor significantly enhance the living standard that compensates for the effects on health resulting from labor activities. In the cold store work environment, there are elements of physical loads, such as noise, vibrations, heat, cold, humidity, and illumination. These elements, when interacting with the worker’s body, cause complex processes, unleashing mechanisms of adaptation, for instance, irritation, sweating, cold and allergic reactions^(13)^. This may explain why 93.3% of the participants in this research stated that they were dissatisfied and 70.1% stated that the work environment is bad.

The discussion brought here addresses the general, particular and singular processes presented by Breilh^(12)^, which aims to discuss how certain social groups are inserted in the production means, the possible effects on their health resulting from labor activities of these specific groups and the wage differences, and living standard. Capitalist production, when adding more value created by workers, makes possible the appearance of a spiral of economic value accumulation (capital accumulation) that coexists with a spiral of exploration, accumulation of poverty, and penetration into new spheres of social life^(14)^. At the same time that the workstations offer stability in the formal market to immigrants, the impact on their health must be considered, having in mind their routine and different workstations that demand strength and repetition in their execution. As seen in the research results, 74.3% of participants work for more than 8 hours a day, generally from Monday to Saturday, with one day to rest that is due to the low salaries the immigrants carry out extra working hours, extending their work journey or working in their rest day. Work accident occurrence is mainly due to activities developed that demand repetitive actions of physical effort.

The work carried out by immigrants allows minimum conditions for their subsistence and causes dissatisfaction situations due to the working conditions and salaries that do not enable them to execute their personal projects. Despite that, the salary derived from Haitian immigrants’ labor activities help to sustain the economy of their country of origin through sending remittances to relatives, however, at the same time, the local economy through expenses with rent and purchase of basic necessities products. A study^(15)^ carried out regarding Haitian immigrants in Brazil highlights that most part of the immigrant workers’ salary is transformed into remittances to their relatives in the country of origin, a situation that reflects the transnational experiences that transcend the State-nation and is configured in a complex social field of migration. Consumption in the capitalist system generates challenges for the underprivileged social classes in the adequacy of their expenses in relation to the salary received^(16)^.

Authors from the field of critical epidemiology^(12,16)^ defend that the empirical evidence of the historical character of the disease must not be searched in individual characteristics but in the processes that occur in the collectivity, that is, the living conditions are produced collectively, and in this same process of production generates social and power relations that determine the distribution of the asset system of which the social reproduction depends on.

The most vulnerable situation of immigrants observed in this research is the difficult adaptation and integration of the receiving country and the bad working conditions associated with racism in the case of Haitians, which increases the risk to health. The data reveal that the work occupies the majority of the time of the interviewed immigrant Haitians; however, as populational groups with different cultural processes, they need to manifest their culture and carry out the integration into the local society. We observed from the data of the research the non- participation of the interviewees in unions and labor organizations, but churches constitute a space of cultural manifestation where immigrant groups meet themselves to discuss daily life experiences, create support networks and share memories from the country of origin, besides being a source of information. The formation of immigrant groups for recreational games or sharing of experiences creates a support network and solidarity. A study carried out regarding Haitian immigrants in Campinas highlighted the central role of religion in the continuity of social integration in the local society^(17)^. The interviewees affirmed that they use the churches to express culture through native country festivals, such as flag parties and immigrant singing choirs. These aspects of shared culture allow socialization and integration with the local society despite the several challenges faced. A study^(9)^ about Haitian immigrants highlighted the different challenges for them to integrate with local society, especially in access to the formal market.

The interviewees evaluated the Unified Health System (SUS) positively, highlighting women that search for more health services than men, accompanied by their children or during pregnancy. The health conditions are related to the routine and work stations, that in the case of cold stores, demand constant physical strength. The symptoms such as pain in the hands; arms, and spine; stress; freezing cold; flu, etc. still do not configure occupational illnesses but are indicative of the way that the health-work relation in cold stores assumes. Besides the aspects related to labor activity, the effects of immigration itself on health must be considered, given the vulnerability context that affects immigrants^(18)^.

Most interviewed immigrants do not carry out health promotion and prevention measures, which is similar to data from a study carried out in Cuiabá, where the most used health services by Haitian immigrants were urgency and emergency^(19)^. Several may be the causes of suffering of Haitian immigrants, the necessity to work extra hours to improve the income, the absence of relatives left in the country of origin, to the lack of integration and access to social policies that generate pressure, stress with effect over the physical and mental health. Although the majority of the interviewed immigrants are young adults with good health conditions, the work conditions evaluated as very bad, associated with the lack of activities on health promotion, the difficulty to access better jobs that allow better living conditions may cause health problems in the short and long term, physical such as psychological.

As limitations of the study, we point out: the impossibility of observing the work process in companies and conducting interviews with those responsible for the sectors that employ immigrants. Hence, qualitative and observational studies carried out inside companies, aligned with reports from participants of this research, may amplify the analysis necessary to deepen the comprehension of the migratory phenomenon in the Western region of Paraná, and that may be able to contribute to the formulation of public policies that minimize the perverse effects of this process.

## CONCLUSIONS

The research results allowed us to analyze aspects of the ways of living, working, consumption, and health of Haitian immigrant residents in the municipality of Cascavel/Paraná, as well as their difficulties with the immigration process and integration into the local society. Besides the challenges to be inserted in the market, other difficulties and objective limitations are experienced, such as the lack of access to education and training and lack of recognition of their qualification obtained in their country of origin. The discrimination suffered by immigrants, if not openly manifested, is perceived in verbal and nonverbal expressions.

The unhealthy working conditions, associated with extensive working hours, dissatisfaction with work stations, and the distance of relatives left in the country of origin, characterizes the immigrants as a vulnerable group subjected to physical and psychological processes of illness. On the other hand, the solidarity among them, the access to public health services, and the possibility of different ways of cultural manifestation constitute protecting factors that must be recognized and valued in the development of their citizenship.

Haitian immigrants’ arrival facilitation to work in local cold stores, in substitution to the work of Brazilians that are reluctant in staying on unhealthy conditions of these places, may indicate not a humanitarian help but the facilitation of labor exploitation of economic and socially vulnerable groups, holders of good health conditions, once they are a population of young adults. The situation of that population is similar to national social groups inserted in the field of activities (slaughterhouse and civil construction industries), which demands repetitive physical strength, with the process of aging diseases will certainly appear, which characterizes the way of living and working of these groups.
